# Iron overload down-regulates the expression of the HIV-1 Rev cofactor eIF5A in infected T lymphocytes

**DOI:** 10.1186/s12953-017-0126-0

**Published:** 2017-08-04

**Authors:** Carmine Mancone, Alessio Grimaldi, Giulia Refolo, Isabella Abbate, Gabriella Rozera, Dario Benelli, Gian Maria Fimia, Vincenzo Barnaba, Marco Tripodi, Mauro Piacentini, Fabiola Ciccosanti

**Affiliations:** 1 Department of Cellular Biotechnologies and Haematology, Sapienza University of Rome, Via Regina Elena 324, 00161 Rome, Italy; 2 Dipartimento di Medicina Interna e Specialità Mediche, Sapienza Università di Roma, Via Regina Elena 324, 00161 Rome, Italy; 3 Department of Epidemiology, Preclinical Research and Advanced Diagnostics, National Institute for Infectious Diseases L. Spallanzani, IRCCS, via Portuense 292, 00149 Rome, Italy; 4 Department of Biological and Environmental Sciences and Technologies (DiSTeBA), University of Salento, 73100 Lecce, Italy; 5 Department of Biology, University of Rome ‘Tor Vergata’, Rome, Italy

**Keywords:** Iron overload, HIV-1 infection, Spike-in SILAC

## Abstract

**Background:**

Changes in iron metabolism frequently accompany HIV-1 infection. However, while many clinical and in vitro studies report iron overload exacerbates the development of infection, many others have found no correlation. Therefore, the multi-faceted role of iron in HIV-1 infection remains enigmatic.

**Methods:**

RT-qPCR targeting the LTR region, *gag*, *Tat* and *Rev* were performed to measure the levels of viral RNAs in response to iron overload. Spike-in SILAC proteomics comparing i) iron-treated, ii) HIV-1-infected and iii) HIV-1-infected/iron treated T lymphocytes was performed to define modifications in the host cell proteome. Data from quantitative proteomics were integrated with the HIV-1 Human Interaction Database for assessing any viral cofactors modulated by iron overload in infected T lymphocytes.

**Results:**

Here, we demonstrate that the iron overload down-regulates HIV-1 gene expression by decreasing the levels of viral RNAs. In addition, we found that iron overload modulates the expression of many viral cofactors. Among them, the downregulation of the REV cofactor eIF5A may correlate with the iron-induced inhibition of HIV-1 gene expression. Therefore, we demonstrated that eiF5A downregulation by shRNA resulted in a significant decrease of Nef levels, thus hampering HIV-1 replication.

**Conclusions:**

Our study indicates that HIV-1 cofactors influenced by iron metabolism represent potential targets for antiretroviral therapy and suggests eIF5A as a selective target for drug development.

**Electronic supplementary material:**

The online version of this article (doi:10.1186/s12953-017-0126-0) contains supplementary material, which is available to authorized users.

## Background

Despite the progress of anti-retroviral therapies, HIV-1 infection is still a major cause of mortality in the world. The ability of the virus to remain latent in a subpopulation of susceptible cells and the development of drug-resistant variants still remain the major obstacles in HIV-1 eradication [1]. Thus, further investigations on the interplay between the HIV-1 life cycle and inducible cellular pathways are required for developing more efficacious therapeutic approach to counteract the infection.

Many enzymes that play crucial roles in cellular metabolism, such as DNA synthesis, replication, transcription and protein translation, require iron to function [2]. Since the life cycle of HIV-1 is associated with enhanced cellular metabolism, it’s no surprise that efficient viral replication needs an iron-replete host [2]. In the last few years, several studies have attempted to shed light on the interplay between iron status and HIV infection providing conflicting evidences since both the anaemia and iron overload are associated with a poor prognosis. In particular, it has been observed that anaemia in HIV-1 infected patients correlates with a worse outcome [3, 4], while other studies demonstrated that genetic iron overload diseases causes a more rapid progression of HIV infection [5, 6]. With respect to in vitro studies, Weinberg and Weinberg (1995) first described the requirement of iron by the HIV-infected host cell for efficient viral particle generation [7]. Recently, increased cellular iron by ferric ammonium citrate has been associated with increased HIV infection and replication in primary CD4^+^ T cells [8]. Moreover, iron overload by FeSO_4_·7H_2_O induced HIV reverse transcriptase activity in the T-lymphoid cell line (CEMs), while no significant changes were observed in viral protein p24 expression [9]. Iron depletion by chelators displayed inhibitory effects on HIV-1 replication in peripheral blood lymphocytes [9–11]. However, while the treatment of monocytes with iron chelators protects against the NF-κB -induced activation of HIV-1 [12], no change in NF-κB has been observed in response to iron chelators [13]. Thus, the effects of iron status on HIV infection remain unclear, likely reflected by the heterogeneity in iron concentrations in studies to date.

Here, we investigated the effects of heavy iron burden in the HIV-1 infected T lymphocyte cell line (C8166). To induce high cellular iron levels, C8166 cells were treated with FeSO_4_·7H_2_O/8-hydroxyquinolone, previously shown to be non-toxic to cells [14, 15]. Notably, iron overload was found to down regulates the expression of *viral proteins*, as well as the downregulation of viral transcripts. To gain insights into the putative mechanism by which iron overload modulates viral gene expression, we performed a “Spike-in” stable-isotope labeling with amino acids in cell culture (Spike-in SILAC) proteomic analysis comparing i) iron-treated, ii) HIV-1-infected and iii) HIV-1-infected/iron treated cells. Then, by integrating proteomic analysis with the HIV-1 Human Interaction Database (National Center for Biotechnology Information, U.S. National Library of Medicine, NCBI), we sought evidence for a possible iron-induced inhibition of HIV-1 gene expression by the Rev cofactor eIF5A.

## Methods

### Reagents

RPMI 1640 Medium cod. R0883, deferipron (L1) cod. 379409, FeSO4/8-Hydroxyquinoline cod. F8633/cod. 252565, sodium chloride cod. S7653, triton cod. X-100, glycerol cod. G5516, ammonium bicarbonate cod. A6141, acetonitrile cod. 14261, DL-dithiothreitol cod. D5545, iodoacetamide cod. 16125, trifluoroacetic acid cod.302031 and a-cyano-4-hydroxycinnamic acid cod. C2020, and MISSION shRNA Bacterial Glycerol Stock cod. SHCLNG-NM_001970 were from Sigma Aldrich, St. Louis, MO, USA. Tris cod. 10708976 were from Roche, Basel - Swiss. NuPAGE 4–12% bis-tris gel cod. NP0335box, SILAC protein quantitation kit cod. 89982 and simply blue safe stain cod. LC6065, with Trizol reagent cod. 15596–018, were from Life technologies, Thermo Fisher Scientific, Waltham, MA, USA. Nitrocellulose membrane Amersham Protan 0,45 μm cod. 10708976 were from GE, Healthcare, Little Chalfont, UK. ECL Luminata classic cod. WBLUC0500, anti Hpu-eif5A cod. ABS1064 and microcolumn ZipTip cod. ZTC185096 were from Millipore Merck, Darmstadt, Germany. Anti-actin antibody cod. sc-1615, anti-calnexin cod.sc-11397 and anti-histone H4 cod. sc-8658 were from Santa Cruz Biotechnology, Dallas, Texas, USA. Anti-Ferritin Heavy chain cod. ab75972, anti HIV-1 Nef cod. ab42355, and anti eif5A cod. ab32014 were from Abcam, Cambridge, UK. Anti-p24 antibody cod. M0857 were from Dako, Agilent Technologies, Santa Clara, CA, USA. Anti-calreticulin cod. ADI-SPA-600 were from Enzo Life Sciences, Farmingdale, NY, USA. Mass Standards kit for Calibration P/N 4333604 were from Sciex, Ontario, Canada. Sequencing grade modified trypsin cod. V5111 were from Promega corporation, Madison, WI, USA.

### HIV-1 in vitro infection and iron overload

C8166 cells were incubated with a HIV-1 pNL4–3 strain (MOI = 1) for 3 h at 37 °C. After washing out unabsorbed virus, cells were cultured in RPMI medium containing 10% FBS. After 24 h cells were treated for the iron overload with FeSO4/8-Hydroxyquinoline 10 uM for 30 min that has been shown to be nontoxic to cells and after 2 h were treated with iron chelator deferipron (L1) 100uM. The cells were lysed after 24 h from the treatment with the iron for the immunoblot detection.

### RNA isolation, cDNA synthesis and RT-qPCR analysis

Cellular RNA was extracted with Trizol reagent accordingly to the manufacturer’s instructions. cDNA synthesis was generated from 2 μg of RNA using the reverse transcription random hexamers method (Promega) according to manufacturer’s recommendations.

Retrotranscribed LTR region was quantified by real-time PCR. After 10 min at 95 °C for enzyme activation, 45 amplification cycles (95° for 10 s, 60 °C for 1 min) were performed (LightCycler® FastStart DNA Master HybProbe – Roche Diagnostics). Total HIV transcripts were quantified by real-time PCR targeting LTR region on 500 ng of retrotranscribed material. cDNA was amplified with the sense primer NEC 152 (GCCTCAATAAAGCTTGCCTTGA) and the reverse primer NEC 131 (GGCGCCACTGCTAGAGATTTT) in the presence of a dually (FAM and TAMRA) labelled NEC LTR probe (AAGTAGTGTGTGCCCGTCTGTTRTKTGACT). As standard curve, dilutions of 8E5 cell DNA containing 1 proviral copy per cell were used.

On the same amount of retrotranscribed material, two specific additional seminested real-time PCR were performed to measure both unspliced RNA (usRNA) and multiply spliced RNA (msRNA).

For usRNA transcripts quantification, a region within the HIV-1 *gag* was amplified. In the first PCR, the primers GAG1 (TCAGCCCAGAAGTAATACCCATGT) and SK431 (TGCTATGTCAGTTCCCCTTGGTTCTCT), with a proof-reading DNA polymerase (Platinum Taq DNA Polymerase High Fidelity, Thermo Fisher Scientific, Milan, Italy), were used. The product of the first PCR underwent as template in the subsequent real-time PCR with GAG1 and GAG2 (CACTGTGTTTAGCATGGTGTTT) as primers, and GAG3 (FAM-ATTATCAGAAGGAGCCACCCCACAAGA-TAMRA) as TaqMan dual-labeled fluorescent probe, on LightCycler® instrument with FastStart DNA Master HybProbe – Roche Diagnostics.

For msRNA transcripts quantification, a region encompassing *Tat* and *Rev.* was amplified. The first PCR was performed with the primer pair ks1 (CTTAGGCATCTCCTATGGCAGGAA) and mf83 (GGATCTGTCTCTGTCTCTCTCTCCACC) using Platinum Taq DNA Polymerase High Fidelity. Subsequently, a real-time PCR was carried out with the primers mf83 and mf84 (ACAGTCAGACTCATCAAGTTTCTCTATCAAAGCA) and the TaqMan fluorescent probe ks2-tq (FAM-TTCCTTCGGGCCTGTCGGGTCCC-TAMRA) on LightCycler® instrument with FastStart DNA Master HybProbe. As standard curve, dilutions of a plasmid containing the product of the first PCR for usRNA and of the second round of PCR for msRNA were used.

### Lentiviral production and infection

Lentiviral particles were produced by transfecting Hek293T cells with the lentiviral vector plko Sh-eif5A or Sh-scramble, together with the Pax2 plasmid (pMDLg/p and pRSV-Rev. plasmids) and ENV (VSV-G) plasmid. Supernatants were collected 48 h post-transfection. The lentiviral suspension was supplemented with polybrene (4 μg/ml, Sigma Aldrich), filtered (PES 0.45 μM filters, Corning) to remove cell debris, and incubated with target cells (2,5 × 10^5^ cells) for 8 h. To increase the transduction efficiency, the infection was repeated twice. After 48 h from infection with lentivirus the cells were infected with HIV-1 and then lysed after 48 h for western blot analysis.

### Immunoblotting analysis

Total cellular proteins were extracted in lysis buffer (150 mM NaCl, 10 mM Tris, 10% glycerol, 0.5% triton).

Twenty micrograms of protein extracts were separated on 4–12% gradient gels by SDS-PAGE and electroblotted onto nitrocellulose membrane. Blots were incubated with primary and secondary antibodies. Antibodies were revealed using ECL. For normalization, membranes were reprobed with an anti-actin antibody.

The following antibodies were used: anti-Ferritin Heavy chain, anti-p24, anti-Nef anti-eif5A, anti-Hpu-eif5A, anti-actin, anti-calnexin, anti-calreticulin, anti-histone H4. Anti-mouse, antirabbit and anti-goat peroxidase conjugated antibodies were from Jackson ImmunoResearch (West Grove, PA, USA).

### SILAC labeling of T lymphocytes cell line (C8166)

C8166 cells and C8166 treated cells (i.e.: iron overload, HIV infection or both conditions) were respectively grown in SILAC “heavy” (^13^C_6_^15^N_4_-arginine and ^13^C_6_-lysine) and SILAC “light” (^12^C_6_^14^N_4_-arginine and ^12^C_6_-lysine) conditions for 8 passages before the experiment. This period lasted about 3 weeks, where the SILAC “heavy” cells labeling was complete. Equal protein amounts (100 μg) of whole cell extracts from C8166 cells and C8166 treated with iron overload, HIV infection or both the conditions were mixed and separated on 4–12% gradient gels by SDS-PAGE.

### Protein digestion, peptide purification, nanoLC analysis, MS analysis

Gels were stained by Simply Blue Safe Stain and sixteen sections for each gel lane were cut. Protein-containing gel pieces were washed with 100 μL of 0.1 M ammonium bicarbonate (5 min at RT). Then, 100 μL of 100% acetonitrile (ACN) was added to each tube and incubated for 5 min at RT. The liquid was discarded, the washing step repeated once more, and the gel plugs were shrunk by adding ACN. The dried gel pieces were reconstituted with 100 μL of 10 mM DTT/0.1 M ammonium bicarbonate and incubated for 40 min at 56 °C for cysteine reduction. The excess liquid was then discarded and cysteines were alkylated with 100 μL of 55 mM IAA/0.1 M ammonium bicarbonate (20 min at RT, in the dark). The liquid was discarded, the washing step was repeated once more, and the gel plugs were shrunk by adding ACN. The dried gel pieces were reconstituted with 12.5 ng/μL trypsin in 50 mM ammonium bicarbonate and digested overnight at 37 °C. The supernatant from the digestion was saved in a fresh tube and 100 μL of 1% TFA/30% ACN were added on the gel pieces for an additional extraction of peptides. The extracted solution and digested mixture were then combined and vacuum centrifuged for organic component evaporation. Peptides were resuspended with 40 μL of 2.5% ACN/0.1% TFA, desalted and filtered through a C18 microcolumn ZipTip and eluted from the C18 bed using 10 μL of 80% ACN/0.1% TFA. The organic component was once again removed by evaporation in a vacuum centrifuge and peptides were resuspended in a suitable nanoLC injection volume (typically 3–10 μL) of 2.5% ACN/0.1% TFA.

An UltiMate 3000 RSLC nano-LC system (Thermo Fisher Scientific, Waltham, MA, USA) equipped with an integrated nanoflow manager and microvacuum degasser was used for peptide separation. The peptides were loaded onto a 75 μm I.D. NanoSeries C18 column (P/N 164534 - Thermo Fisher Scientific, Waltham, MA, USA) for multistep gradient elution (eluent A 0.05% TFA; eluent B 0.04% TFA in 80% ACN) from 5 to 20% eluent B within 10 min, from 20 to 50% eluent B within 45 min and for further 5 min from 50 to 90% eluent B with a constant flow of 0.3 μL/min. After 5 min, the eluted sample fractions were continuously diluted with 1.2 μL/min a-cyano-4-hydroxycinnamic acid (CHCA) and spotted onto a MALDI target using a HTC-xt spotter (PAL SYSTEM) with an interval of 20 s resulting in 168 fractions for each gel slice. Mass Spectrometry Analysis MALDI-TOF-MS spectra were acquired using a 5800 MALDI TOF/TOF Analyzer (Sciex, Ontario, Canada). The spectra were acquired in the positive reflector mode by 20 subspectral accumulations (each consisting of 50 laser shots) in an 800–4000 mass range, focus mass 2100 Da, using a 355 nm Nb:YAG laser with a 20 kV acceleration voltage. Peak labeling was automatically done by 4000 Series Explorer software Version 4.1.0 (Sciex, Ontario, Canada) without any kind of smoothing of peaks or baseline, considering only peaks that exceeded a signal-to noise ratio of 10 (local noise window 200 m/z) and a half maximal width of 2.9 bins. Calibration was performed using default calibration originated by five standard spots. Only MS/MS spectra of preselected peaks (out of peak pairs with a mass difference of 6.02, 10.01, 12.04, 16.03, and 20.02 Da) were integrated over 1000 laser shots in the 1 kV positive ion mode with the metastable suppressor turned on. Air at the medium gas pressure setting (1.25 × 10^−6^ Torr) was used as the collision gas in the CID off mode. After smoothing and baseline subtractions, spectra were generated automatically by 4000 Series Explorer software. MS and MS/MS spectra were processed by ProteinPilot Software 4.5 (Sciex, Ontario, Canada) which acts as an interface between the Oracle database containing raw spectra and a local copy of the MASCOT search engine (Version 2.1 - Matrix Science, Boston, MA, USA). The Paragon algorithm was used with SILAC (Lys + 6, Arg + 10) selected as the Sample Type, iodacetamide as cysteine alkylation, with the search option “biological modifications” checked, and trypsin as the selected enzyme. MS/MS protein identification was performed against the Swiss-Prot database (number of protein sequences: 254,757; released on 20,121,210) without taxon restriction using a confidence threshold of 95% (Proteinpilot Unused score ≥ 1.31). The monoisotopic precursor ion tolerance was set to 0.12 Da and the MS/MS ion tolerance to 0.3 Da. The minimum required peptide length was set to 6 amino acids; two peptides were required for protein identification. For quantitation, the Heavy/Light average ratio for a protein was calculated by ProteinPilot Software with automatic bias correction. Quantitation was based on a two-dimensional centroid of the isotope clusters within each SILAC pair. Ratios of the corresponding isotope forms in the SILAC pair were calculated, and lines fitting these intensity ratios gave the slope as the desired peptide ratio. To represent the ratio of a peptide being quantified several times, the median value was chosen. To minimize the effect of outliers, protein ratios were calculated as the median of all SILAC pair ratios that belonged to peptides contained in this protein. The percentage of quantitation variability was defined as the standard deviation of the natural logarithm of all ratios used for obtaining the protein ratio multiplied by a constant factor of 100.

### Data analysis

Differentially expressed proteins were analyzed using Ingenuity Pathway Analysis (IPA, Ingenuity Systems; see www.ingenuity.com). The over-represented diseases and bio functions annotations were generated based on information contained in the Ingenuity Pathways Knowledge Base. Right-tailed Fisher’s exact test was used to calculate a *p*-value determining the probability that each biological function and/or disease involved in that proteome profile alteration is due to chance alone.

## Results and discussion

### High cellular iron downregulates HIV-1 proteins production and gene expression

**Fig. 1:**
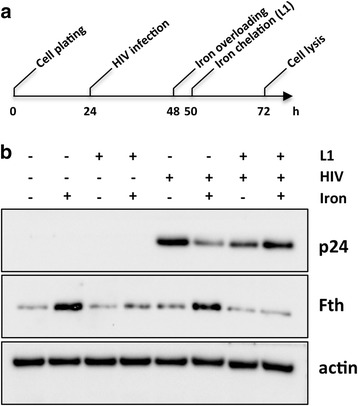
Effects of iron excess on HIV-1 gene expression. **a** Schematic representation of experimental workflow. Timing of treatments is indicated. **b** Immunoblot detection of viral protein p24. Ferritin heavy chain (Fth) expression was used as iron overloading control; actin expression as protein loading control. These images are representative of experiments carried out in triplicate

To ascertain the effects of iron excess on HIV infection, we subjected C8166 cells to iron overload 24 h post-HIV-1 infection (Fig. 1a). The viral gene expression was evaluated by monitoring the levels of the most abundant HIV-1 structural protein (p24). In fact, even if post-transcriptional RNA splicing generates more than 30 different viral RNA species, the viral protein p24 originates only from the translation of 9-kb unspliced genomic transcripts, the only ones encoding the *gag* structural proteins [16, 17]. Surprisingly, as shown in Fig. 1b, high iron cellular levels led to significant decrease of p24 expression. Notably, iron-induced p24 downregulation was abrogated when cellular iron concentrations were normalized by iron depletion treatment with deferiprone (L1), thus suggesting a direct correlation between iron concentration and viral gene expression (Fig. 1a and b). Moreover, we extended our observations on HIV-1 non structural protein Nef, that is encoded by the 2-kb completely spliced mRNA. Interestingly, the expression of Nef was reduced in presence of iron overload (Additional file 1), thus indicating that the iron excess lead to a general viral proteins downregulation.

**Fig. 2:**
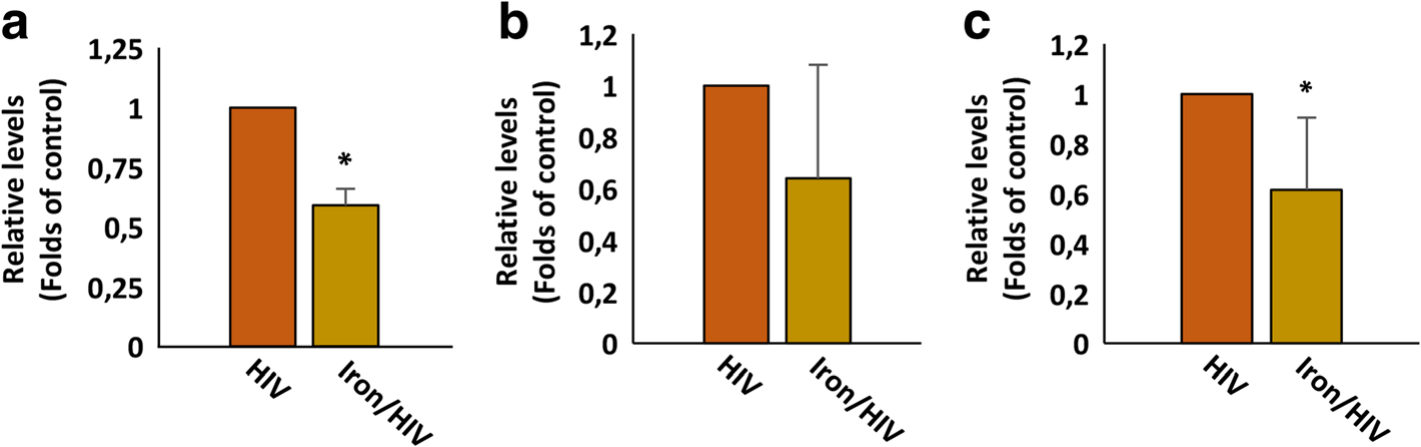
Effects of iron excess on HIV-1 transcripts levels. Levels of total (**a**), unspliced (**b**) and multi-spliced (**c**) viral RNAs were measured 48 h post infection by quantitative real-time PCR (qPCR) in cells with and without iron overload treatment. Results are average of three independent experiments ± SD

Next, we evaluated whether iron overload affects viral gene expression at the translational level. To this aim RT-qPCR targeting the LTR region, *gag*, *Tat* and *Rev* were performed in order to measure the levels of total, unspliced and multispliced viral RNAs in response to iron overload. As shown in Fig. 2, HIV-1-infected cells expressed significantly lower levels of viral RNAs when undergoing iron overload.

Taken together these data demonstrate that high iron cellular concentration inhibits HIV-1 life cycle in T lymphocytes by downregulating viral RNA expression.

### Iron overload significantly modifies the proteome profile of HIV-1-infected T lymphocytes

As with all viruses, HIV-1 is strictly dependent on the host cell to obtain energy, macromolecules and the structural organization necessary for its replication. Therefore, modifications in the host cell proteome occurring in response to iron overload may unveil host factors involved in the restriction of the HIV-1 infection.

**Fig. 3:**
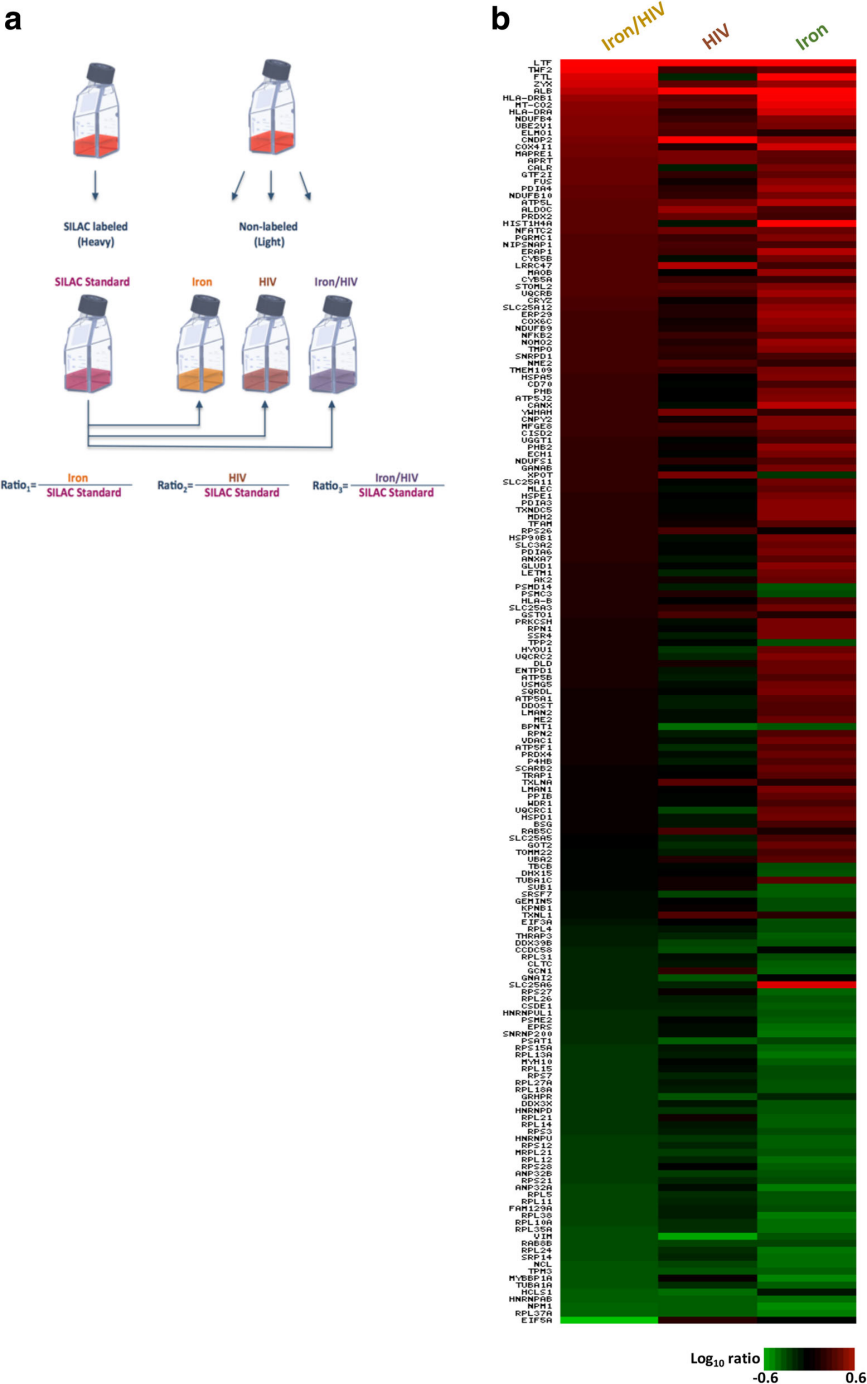
Spike in SILAC workflow. **a** Spike-in SILAC standard labeling (C8166 Heavy) was “decoupled” from the biological experiments and then carried out under normal cell culture conditions (C8166 Light). After the treatments were performed, the nonlabeled samples were combined with the SILAC standard and each of these combined samples was analysed separately by LC-MS/MS. Ratio 1 originated from the light/heavy ratio between iron-overloaded cells (Fe) and SILAC standard; Ratio 2 from HIV-infected cells (HIV) and SILAC standard; Ratio 3 from iron-overloaded and HIV-infected cells (HIV/Fe) and SILAC standard. **b** Iron overload perturbs the proteome profile of the HIV-infected cells. Each vertical column represents an individual condition and each horizontal row an individual protein; gene names are indicated. Protein abundance ratios were colored according to the fold changes (*green* Log10 ratios: downregulations; *red* Log10 ratios: upregulations) and the color scale indicates the magnitude of expression changes. *Black* squares indicate no change in protein abundance. Proteins exhibiting abundant changes ≥1.5-fold increase in at least one of the three conditions were reported

The Spike-in SILAC approach has proven to be an excellent strategy for multiple quantitative comparisons among samples [15, 18]. Here, for the Spike-in SILAC internal standard production, C8166 cells were metabolically labeled with ^13^C_6_^15^N_4_-arginine and ^13^C_6_-lysine (Heavy medium). Non-labeled cell populations were firstly grown in light medium (^12^C_6_^14^N_4_-arginine and ^12^C_6_-lysine) and subsequently subjected to iron overload (Iron), viral infection (HIV) or both (Iron/HIV) (Fig. 3a). Whole cell extracts were isolated separately and each non labelled sample combined with equal amounts of SILAC standard cell extract prior to subjection to nanoLC-MALDI-TOF/TOF analysis and resulting in the identification of 644 proteins differently expressed in each treatment condition (Additional file 2). To validate the proteomics results, Spike-in SILAC ratios of calnexin, calreticulin and histone H4 were further confirmed by western blotting. In line with the SILAC data, the levels of these proteins were found to be increased in Iron-treated cells with respect to those of HIV and Iron/HIV conditions (Additional file 3), thus corroborating the proteomic observations.

**Fig. 4:**
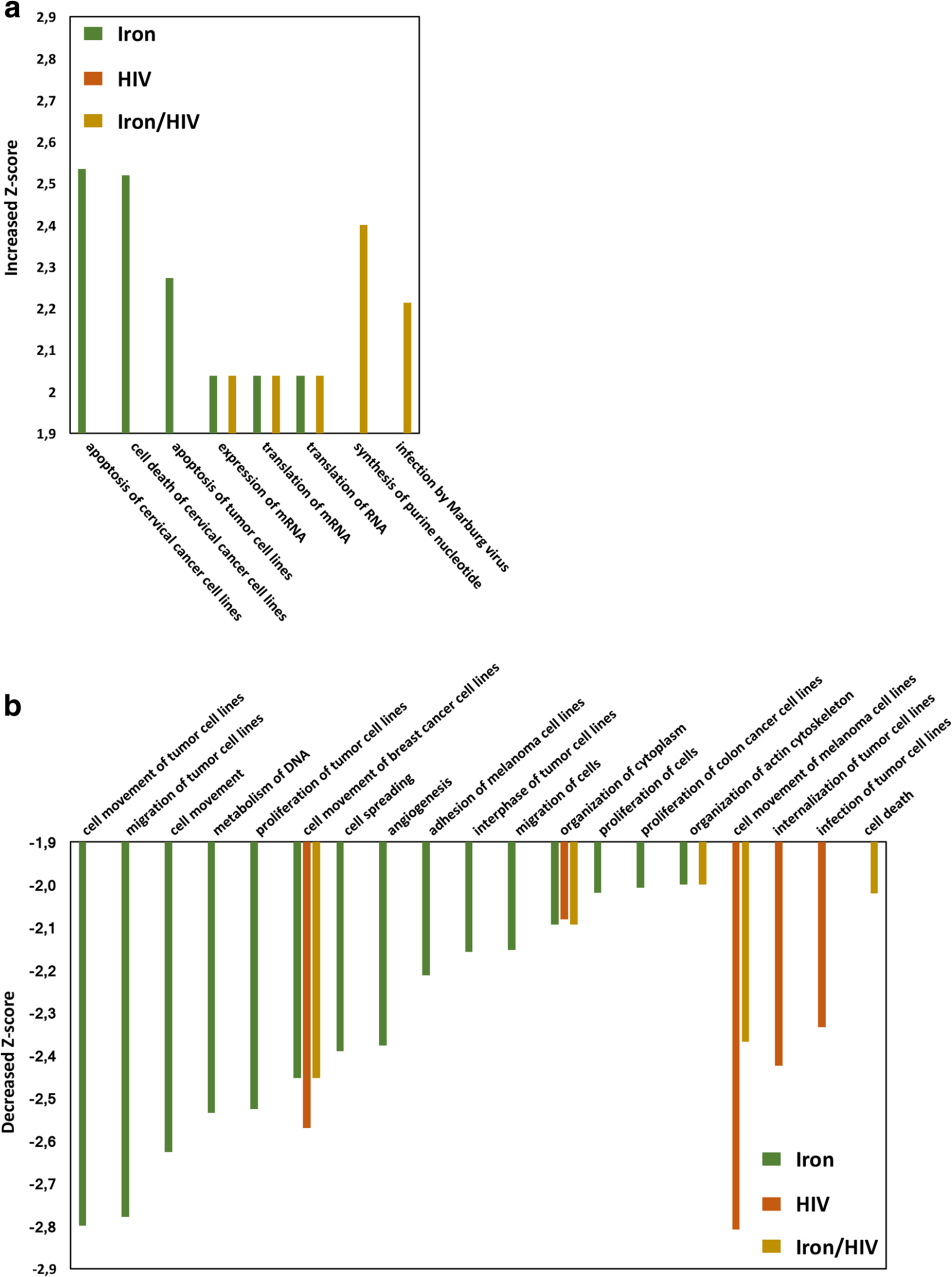
Diseases and bio functions analysis. IPA activation Z-scores (Y-axes) for the indicated annotations (X-axes) in Iron, HIV and Iron/HIV conditions were summarized as histograms. Z-score thresholds were selected >2 for increased (**a**) and <−2 for decreased (**b**) predicted activation states; only annotations with *p*-value <0,05 were reported

We then focused on proteins exhibiting abundant changes ≥1.5-fold. In HIV-1 infected cells, these proteins account for 6,2% of the total. Instead, heavy iron overload significantly altered the C8166 proteome profile by modulating 27,7% of identified proteins. Surprisingly, the percentage of modulated proteins in HIV-1 infected cells with iron overloading was found to be significantly lower (8,5%) with the respect to the Iron only condition. In light of these differences, protein expression levels of HIV-1 infected cells with iron overload (Iron/HIV) were compared by heat map with those subjected to iron over load or HIV infection alone of the two conditions separately (Matrix2png, version 1.2.2). As shown in Fig. 3b, the heat map pattern of Iron/HIV treated cells revealed significant differences when compared to the Iron and HIV single profiles. The peculiarity of Iron/HIV proteome profile was further confirmed by a bioinformatic analysis. In fact, to unveil the molecular functions underlying the effects of iron excess on HIV-1 infected cells, identified proteins were functionally grouped according to the Ingenuity Pathways Analysis literature database. All the differentially expressed proteins were therefore uploaded to the IPA server and the three proteomic data sets (Iron, HIV, Iron/HIV) were analysed in the frame of diseases and functions. As shown in Fig. 4, iron overload in C8166 was responsible for the most increased and decreased listed functions. On the contrary, the iron overload in the frame of HIV-1 infection resulted in an unedited and most attenuated profile when compared to the Iron and HIV single profiles. Interestingly, in iron overloaded cells, the modulation of proteins involved in the apoptotic process generated an increased activation state (Fig. 4a), while in the Iron/HIV cells the apoptosis was predicted as down-regulated (Fig. 4b).

Taken together, these data highlight that iron overload in comorbidity with viral infection leads to the generation of a peculiar host protein response.

### Iron-induced downregulation of the HIV-1 Rev cofactor eIF5A

The intimate interactions between viral proteins and host cellular factors are absolutely required for the 9-kb unspliced genomic transcript expression which in turn is mandatory for HIV-1 replication. Therefore, to gain insight into iron-induced inhibition of HIV-1 genomic RNA expression, we analysed the expression protein profiles of HIV cofactors in Iron/HIV cells. To this aim, the Iron/HIV proteomic dataset was matched with the HIV-1 Human Interaction Database (NCBI), which collates hundreds of reports of protein interactions that affect viral replication and infectivity [19–21]. In this way, the expression levels of four viral cofactors (eIF5A, UBE2V1, HLA-DRA, HLA-DRB1) were found to impact on HIV-1 life cycles (Additional file 4). However, only the downregulation of eukaryotic translation initiation factor 5A-1 (eIF5A) was associated with the decreased levels of 9-kb unspliced genomic transcripts.

**Fig. 5:**
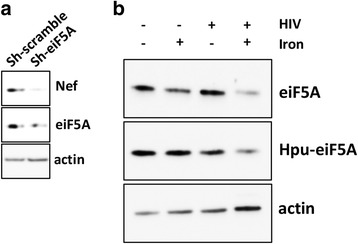
eiF5A downregulation and HIV-1 infection. **a** Western blotting analysis for eiF5A and Nef was performed on HIV-1-infected c8166 cells with either scrambled control shRNA or shRNA targeting eif5A. **b** eiF5A is downregulated in HIV-infected cells with iron overload. eiF5A and its hypusinated form (Hpu-eif5A) were detected by immunoblot. Actin expression was used as loading control. One representative experiment out of three is shown

eIF5A is a translation factor that plays a role in both initiation and elongation [22]. Additionally, it is implicated in transcription, mRNA turnover and nucleocytoplasmic transport [22]. eIF5A is the only cellular protein known to contain the unusual amino acid hypusine, a modification that is required for cell proliferation and viability [23]. In HIV-1 infection, hypusinated eIF5A is a HIV-1 Rev interacting protein essential for the viral unspliced genomic RNA nuclear export [24]. In fact, it has been extensively demonstrated that down modulation, mutations and the inhibition of hypusination of eIF5A block the nuclear export of Rev proteins, thus inhibiting the translocation of 9-kb unspliced genomic RNA and consequently prevent the HIV-1 replication [24–28]. Here, to confirm in our working model the pivotal role of eiF5A in HIV-1 replication, the expression of eiF5A was silenced by shRNA. As shown in Fig. 5a, eiF5A downregulation resulted in a significant decrease of Nef levels. Interestingly, in the iron treated/HIV infected cells, the downregulation of eIF5A was an effect of comorbidity more than an iron-mediated consequence (Additional file 2 and Fig. 5b). Moreover, immunoblot detection of hypusine showed the same protein pattern, thus indicating that iron overload does not affect the modification of eIF5A.

## Conclusions

In this report, we describe the effects of iron overload on HIV-1 infected T lymphocytes in vitro. Firstly, our results indicate that iron excess leads to the down regulation of the HIV-1 gene expression of viral proteins at a pre-translational level, thus restricting viral infection. We believe that these findings contribute to the elucidation of the multi-faceted role of iron metabolism in HIV infections. However, since the present study focused on considering that FeSO_4_·7H_2_O/8-hydroxyquinolon pulse in C8166 cells, further experimentation in animal models are needed to establish the in vivo potential significance of these results.

Interestingly, quantitative proteomics comparing iron-treated, HIV-1-infected and HIV-1-infected/iron treated cells, followed by data mining with the HIV-1 Human Interaction Database led to definition of a possible mechanism for the iron-mediated effect. In fact, it appears conceivable that the correlation between iron-induced eIF5A downregulation and the restriction of HIV-infection has a causal implication: the iron-induced restriction of HIV infection may arise from the inhibition of genomic RNA nuclear export, as a result of iron-induced eIF5A downregulation, thus leading to prevent the HIV-1 replication. Although this hypothesis remains to be confirmed, our study further indicates that HIV-1 cofactors influenced by iron metabolism represent potential targets for antiretroviral therapy.

In keeping with this assumption previous studies have demonstrated the pharmacological inhibition of eIF5A biosynthesis represents an important candidate for the therapy of HIV infection [29, 30]. Based on the considerable therapeutic interest in eIF5A as a selective target for drug development, the iron effect described in this study acquires particular relevance and should be taken into full consideration for clinical trials.

## Additional files

Additional file 1: Immunoblot detection of viral protein Nef. Actin expression was used as protein loading control. This image is representative of experiments carried out in triplicate. (DOCX 1588 kb)

Additional file 2: Densitometric analysis of calnexin, calreticulin and histone H4 levels in the indicated conditions in respect to Silac Standard. Twenty micrograms of protein extracts from SILAC preparations were separated on 4–12% gradient gels by SDS-PAGE and electroblotted onto nitrocellulose membrane. The chemiluminescent blots were imaged with the ChemiDoc MP imager (Bio-Rad) and the band analysis tools of ImageLab software version 4.1 (Bio-Rad) were used to select and determine the background-subtracted density of the bands in all blots. Protein bands from calnexin, calreticulin and H4 immunoblots were normalized by actin expression. Obtained ratios were reported as histograms. (DOCX 5689 kb)

Additional file 3: Analysis of proteomic data by HIV-1 Human Interaction NCBI Database. Up- and down-regulated proteins of Iron/HIV proteomic dataset exhibiting abundance changes ≥2-fold increase were considered. (DOCX 80 kb)

Additional file 4: Integration of data from quantitative proteomics with the HIV-1 Human Interaction Database. (XLSX 250 kb)
