# Supplementary material for: Iron overload down-regulates the expression of the HIV-1 Rev cofactor eIF5A in infected T lymphocytes
Source: Proteome Sci. 2017 Aug 4;15:18. doi: 10.1186/s12953-017-0126-0 (PMC5545036; doi:10.1186/s12953-017-0126-0)
Supplement: Supplementary file 1 — Immunoblot detection of viral protein Nef. Actin expression was used as protein loading control. This image is representative of experiments carried out in triplicate. (DOCX 1588 kb) [file 12953_2017_126_MOESM1_ESM.docx]

Additional File 1


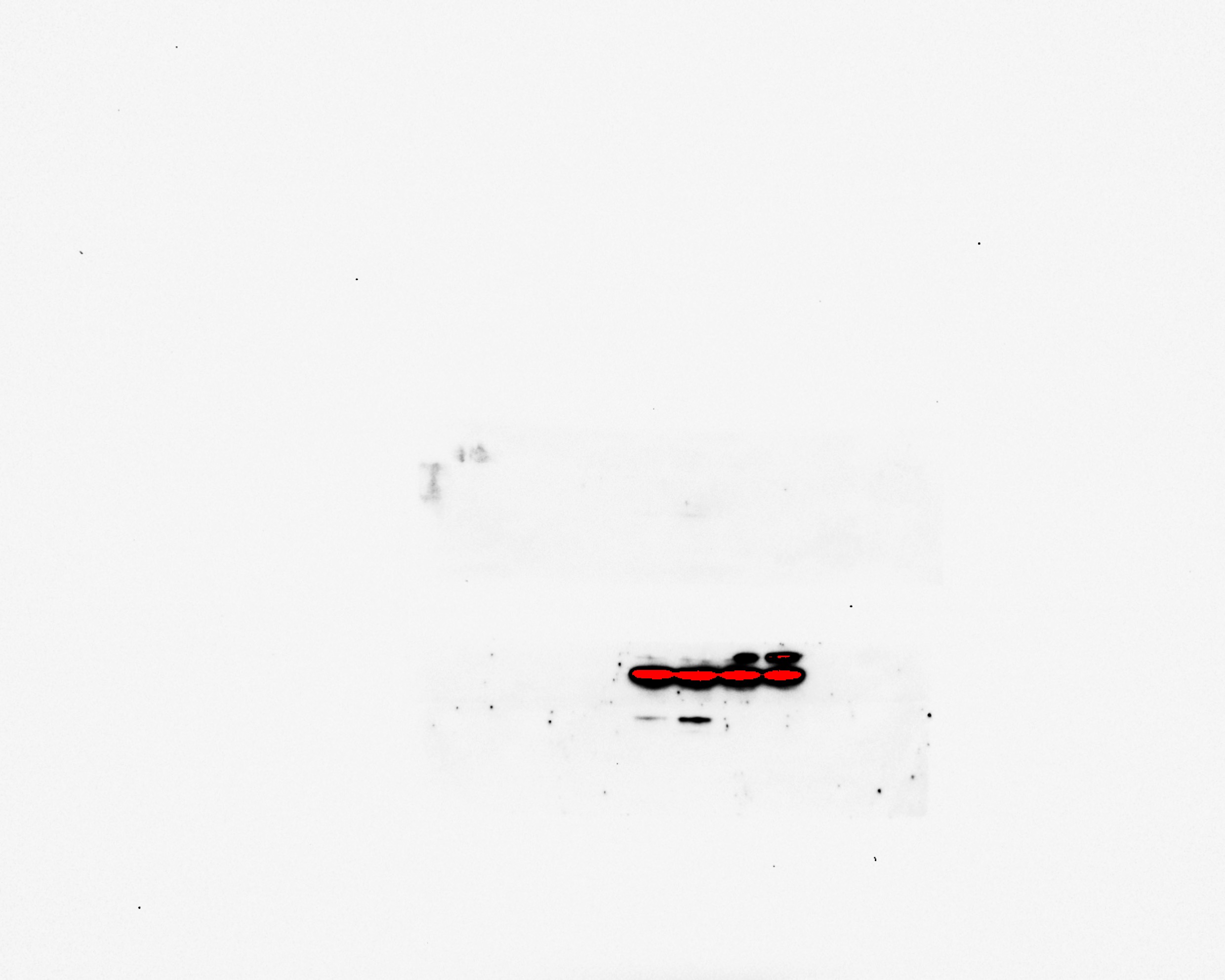


**Nef**

**HIV**

**Iron/HIV**


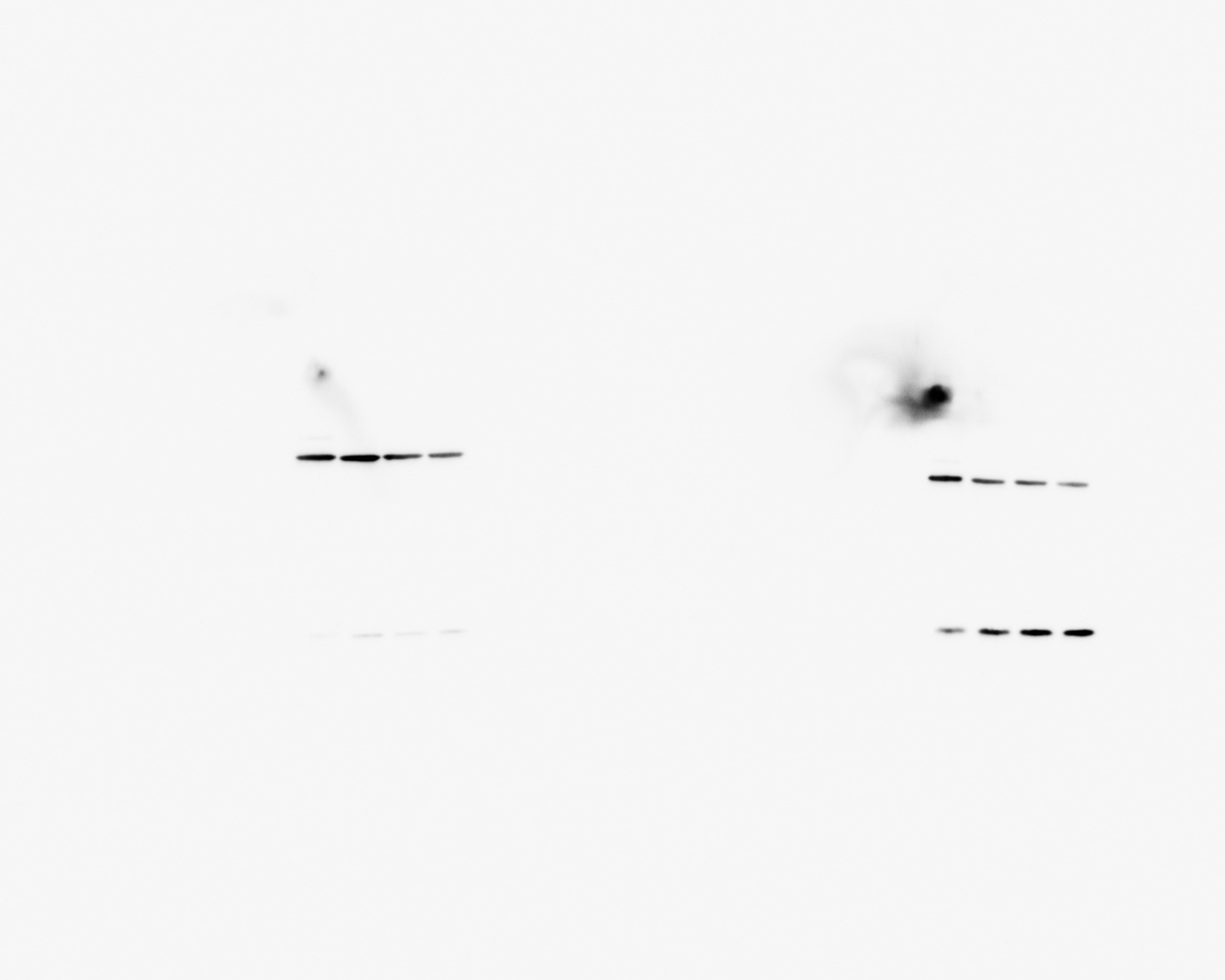


**actin**

**Immunoblot detection of viral protein Nef**. Actin expression was used as protein loading control. This image is representative of experiments carried out in triplicate.
