# Supplementary material for: Iron overload down-regulates the expression of the HIV-1 Rev cofactor eIF5A in infected T lymphocytes
Source: Proteome Sci. 2017 Aug 4;15:18. doi: 10.1186/s12953-017-0126-0 (PMC5545036; doi:10.1186/s12953-017-0126-0)
Supplement: Supplementary file 2 — Densitometric analysis of calnexin, calreticulin and histone H4 levels in the indicated conditions in respect to Silac Standard. Twenty micrograms of protein extracts from SILAC preparations were separated on 4–12% gradient gels by SDS-PAGE and electroblotted onto nitrocellulose membrane. The chemiluminescent blots were imaged with the ChemiDoc MP imager (Bio-Rad) and the band analysis tools of ImageLab software version 4.1 (Bio-Rad) were used to select and determine the background-subtracted density of the bands in all blots. Protein bands from calnexin, calreticulin and H4 immunoblots were normalized by actin expression. Obtained ratios were reported as histograms. (DOCX 5689 kb) [file 12953_2017_126_MOESM2_ESM.docx]

Additional File 2


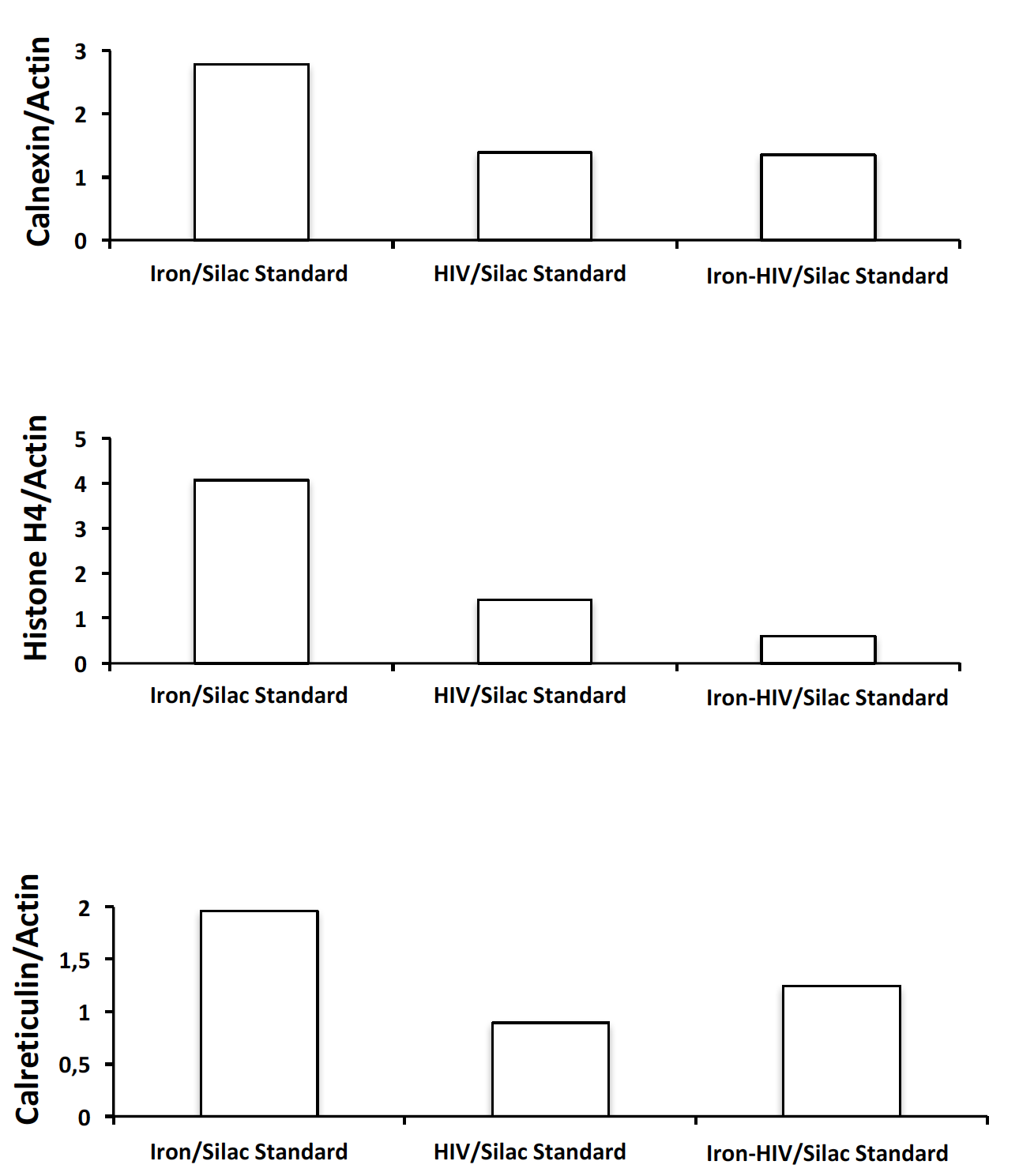


**Densitometric analysis of calnexin, calreticulin and histone H4 levels in the indicated conditions in respect to Silac Standard**. Twenty micrograms of protein extracts from SILAC preparations were separated on 4−12% gradient gels by SDS-PAGE and electroblotted onto nitrocellulose membrane. The chemiluminescent blots were imaged with the ChemiDoc MP imager (Bio-Rad) and the band analysis tools of ImageLab software version 4.1 (Bio-Rad) were used to select and determine the background-subtracted density of the bands in all blots. Protein bands from calnexin, calreticulin and H4 immunoblots were normalized by actin expression. Obtained ratios were reported as histograms.
