# Supplementary material for: Iron overload down-regulates the expression of the HIV-1 Rev cofactor eIF5A in infected T lymphocytes
Source: Proteome Sci. 2017 Aug 4;15:18. doi: 10.1186/s12953-017-0126-0 (PMC5545036; doi:10.1186/s12953-017-0126-0)
Supplement: Supplementary file 3 — Analysis of proteomic data by HIV-1 Human Interaction NCBI Database. Up- and down-regulated proteins of Iron/HIV proteomic dataset exhibiting abundance changes ≥2-fold increase were considered. (DOCX 80 kb) [file 12953_2017_126_MOESM3_ESM.docx]

| **Host protein** | **Iron/HIV host protein regulation (Fold Change)** | **Interaction with HIV protein** | **Effect of host protein regulation on** | |
| --- | --- | --- | --- | --- |
|  |  |  | **HIV life cycle** | **Viral unspliced RNA expression** |
| EIF5A | -2,8 | Nef | / | / |
|  |  | Rev | Downregulation | Downregulation |
| CNDP2 | 2,0 | / | / | / |
| ELMO1 | 2,0 | Nef | / | / |
| UBE2V1 | 2,0 | Capsid | Downregulation | / |
|  |  | Tat | / | / |
| NDUFB4 | 2,1 | / | / | / |
| HLA-DRA | 2,1 | Gp120 | / | / |
|  |  | Gp160 | / | / |
|  |  | Gp41 | / | / |
|  |  | Nef | / | / |
|  |  | Pr55(Gag) | Upregulation | / |
|  |  | Tat | / | / |
|  |  | Vpu | / | / |
|  |  | Capsid | / | / |
| MT-CO2 | 2,1 | / | / | / |
| HLA-DRB1 | 2,3 | Gp120 | / | / |
|  |  | Gp160 | / | / |
|  |  | Gp41 | / | / |
|  |  | Nef | / | / |
|  |  | Pr55(Gag) | Upregulation | / |
|  |  | Tat | / | / |
|  |  | Vif | / | / |
|  |  | Vpu | / | / |
|  |  | Capsid | / | / |
| ZYX | 3,1 | Nef | / | / |
| FTL | 3,2 | Nef | / | / |
| TWF2 | 3,8 | / | / | / |

**Additional file 3. Analysis of proteomic data by HIV-1 Human Interaction NCBI Database.** Up- and down-regulated proteins of Iron/HIV proteomic dataset exhibiting abundance changes ≥2-fold increase were considered.
